# Conserved tripartite tail proteins mediate virophage-host interactions through *Synechococcus* lipopolysaccharide binding

**DOI:** 10.1128/jvi.01082-25

**Published:** 2025-11-18

**Authors:** Ting Chu, Qinran Wang, Chen Hu, Zexian Zhou, Bo Li, Yesheng Yan, Xiang Li, Aohan Wang, Qianglin Fang, Shuling Yan, Lanming Chen, Yongxin Yu, Yongjie Wang

**Affiliations:** 1 College of Food Science and Technology, Shanghai Ocean University 74595, Shanghai, China; 2 Laboratory for Marine Biology and Biotechnology, Qingdao Marine Science and Technology Center 554912 , Qingdao, China; 3 Shanghai Engineering Research Center of Food Microbiology, School of Health Science and Engineering, University of Shanghai for Science and Technology 47863, Shanghai, China; 4 School of Public Health (Shenzhen), Shenzhen Campus of Sun Yat-sen University 660329 , Shenzhen, Guangdong, China; 5 Entwicklungsgenetik und Zellbiologie der Tiere, Philipps-Universität Marburg 9377, Marburg, Germany; Cornell University Baker Institute for Animal Health, Ithaca, New York, USA

**Keywords:** virophage, tail fiber protein, heterotrimer, receptor binding, alga, cyanobacteria

## Abstract

Virophages are small double-stranded DNA viruses that parasitize giant viruses and play crucial roles in microbial ecosystem dynamics. In this study, we investigate three evolutionarily conserved tail proteins in Dishui Lake Virophage 2 (DSLV2) that exhibit significant structural homology to the T4 phage gp37 long tail fiber protein. Our integrated approach combining structural modeling, molecular dynamics simulations, and biochemical characterization reveals that these proteins form a stable heterotrimeric complex with a functional receptor-binding domain. Comprehensive genomic analysis of related virophages within the Aquatic Virophage 1 lineage demonstrates the widespread conservation of this tripartite tail architecture, suggesting its fundamental importance in virophage biology. Phylogenetic evidence indicates that these tail proteins of DSLV2 were evolutionarily related to those of *Synechococcus* phages, and *Synechococcus* cell surface lipopolysaccharides (LPSs) serve as receptors for the binding of the heterotrimeric tails. We propose a novel two-step infection mechanism wherein DSLV2 initially attaches to *Synechococcus* LPSs via its heterotrimeric tail complex, followed by subsequent entry into algal cells through bacterivory-mediated viral hitchhiking. These findings significantly advance our understanding of virophage-host interaction mechanisms in aquatic ecosystems.

**IMPORTANCE:**

This study significantly advances our understanding of virophage biology by elucidating key molecular mechanisms underlying their host interactions. The discovery of a conserved heterotrimeric tail complex in Dishui Lake Virophage 2, evolutionarily related to tail proteins of *Synechococcus* phages and specifically recognizing *Synechococcus* lipopolysaccharide (LPS), reveals an unexpected life cycle for virophages and provides the first structural basis for their attachment strategy. The proposed two-step infection mechanism involving initial LPS binding followed by bacterivory-mediated algal cell entry challenges conventional views of virophage entry and suggests that these viruses may play a more complex ecological role than previously recognized. These findings not only shed light on the evolutionary adaptation of virophages but also have important implications for understanding complex microbial interactions in aquatic ecosystems, particularly in terms of bacteria-mediated virophage cycling.

## INTRODUCTION

Virophages (the class *Virophaviricetes*) are small, non-enveloped double-stranded DNA (dsDNA) viruses with genomes spanning 17–42 kilobase pairs (kb) and icosahedral capsids measuring 50–75 nm in diameter (1–3). These viruses establish a hyperparasitic relationship with giant viruses and eukaryotic hosts, forming a tripartite cell−giant virus−virophage (CVv) system (4–11). By hijacking the replication machinery of giant viruses, virophages suppress their propagation, thereby reducing host cell mortality (5, 6, 8, 12). Metagenomic studies have uncovered their global diversity and ecological prevalence, highlighting their widespread distribution across diverse habitats (13–23). The CVv interaction network plays a crucial role in shaping eukaryotic cell biology and evolution while also modulating ecosystem dynamics—particularly in microbial protist communities—by regulating giant virus-induced host mortality (15, 24–26).

The identification of CVv systems involving amoebas, flagellates, and algae as eukaryotic hosts has been enabled through co-culture techniques and metagenomic approaches (5, 6, 16–19, 25, 27). Two well-characterized examples include (i) the pioneering *Acanthamoeba*-APMV-Sputnik system, where Sputnik virophages attach to glycosylated fibers on the *Acanthamoeba polyphaga* mimivirus (APMV) capsid and enter host cells via co-phagocytosis (5, 28–31); and (ii) the *Cafeteria*-CroV-Mavirus system, where Mavirus virophages independently enter flagellate *Cafeteria burkhardae* through receptor-mediated endocytosis and can integrate into the host genome prior to *Cafeteria roenbergensis* virus (CroV) infection (4, 6, 8). Apparently, critical knowledge gaps remain regarding (i) the molecular mechanisms of virophage recognition of host cells or giant viruses, (ii) the identification and characterization of virophage receptor-binding proteins (RBPs), and (iii) the replication cycles and entry pathways in algal-hosted CVv systems (27).

In contrast to virophage RBPs, phage RBPs have been extensively characterized in terms of structure and function. For instance, the T4 phage tail employs long tail fibers (gp34–gp37) and short tail fibers (gp12) as RBPs (32). During infection, the long tail fibers dynamically switch between folded and extended conformations to initiate binding to host receptors, such as lipopolysaccharides (LPSs) or outer membrane proteins (e.g., OmpC) (33, 34). Unlike phages, which rely on mechanized tail structures for host penetration, virophages may depend primarily on highly specific RBP-receptor interactions for adhesion (29, 35).

Recently, we identified three consecutive tail protein-encoding genes in Dishui Lake Virophage 2 (DSLV2) (19). The distinctive genomic arrangement and abundance of these genes suggest their potential role as RBPs, analogous to phage tail proteins. To investigate this, we performed comprehensive structural and functional analyses, revealing that these proteins assemble into a heterotrimeric complex. This complex targets cyanobacterial LPS, likely facilitating virophage entry into host cells through algal bacterivory. Our findings provide new insights into the dissemination mechanisms of virophages involved in alga-giant virus-virophage tripartite systems.

## MATERIALS AND METHODS

### Protein structure prediction and comparison

The monomeric structures of three consecutive DSLV2 tail proteins (QIG59353.1, QIG59354.1, QIG59355.1) were independently predicted using the online AlphaFold3 server (https://golgi.sandbox.google.com/) (36). To evaluate structural similarities with known protein structures, we conducted structural alignments through the DALI server (http://ekhidna2.biocenter.helsinki.fi/dali/) (37). In DALI alignment results, the Z-score serves as a crucial metric for structural similarity, where values exceeding 2 generally denote significant structural resemblance between compared proteins. These structural relationships were further visualized using DALI-generated similarity matrices that compared each target structure against its top-matched reference structure.

For higher-order structure prediction, both trimeric assemblies and protein complexes were modeled using AlphaFold3 (36). The reliability of these predicted structures was quantified through two key metrics: the predicted template modeling (pTM) score and the interface predicted template modeling (ipTM) score. The pTM score, reflecting overall structural accuracy, considers predictions with scores above 0.5 to exhibit high similarity to native folds. The ipTM score specifically evaluates subunit arrangement in complexes, with confidence thresholds defined as follows: scores > 0.8 indicate high prediction confidence, scores < 0.6 suggest probable prediction failure, while scores between 0.6 and 0.8 represent an intermediate confidence range where prediction accuracy remains uncertain.

### Conserved domain analysis

To characterize the domain architecture of DSLV2’s three consecutive tail proteins, we performed sequence alignment against the gp37 reference sequence (residues 785–1,026). Residues 811–860 and 1016–1,026 of gp37 constitute the trimeric collar domain, while residues 907–996 form the receptor-binding domain (38). Additionally, we systematically identified domains across 135 virophage tail proteins using Batch CD-Search (https://www.ncbi.nlm.nih.gov/Structure/bwrpsb/bwrpsb.cgi) (39), enabling comprehensive structural comparisons.

### Molecular dynamics simulation

Molecular dynamics (MD) simulations were performed using GROMACS 2025.1 (https://www.gromacs.org) (40). The protein receptor’s topology was generated via the “--pdb2gmx” command, and the Amber ff14SB force field (https://ambermd.org/ForceFields/ff14sb) was employed for system parameterization. The complex was embedded in a rhombic dodecahedron box, solvated with TIP3P water, and neutralized with 0.15 M NaCl to approximate physiological ionic strength. Counterions were added to ensure charge neutrality.

System equilibration proceeded in two phases: (i) NVT ensemble (constant particle number, volume, and temperature) with positional restraints, gradually heating the system to 300 K using the V-rescale thermostat, and (ii) NPT ensemble (constant particle number, pressure, and temperature) with isotropic pressure coupling (C-rescale), during which positional restraints were incrementally relaxed.

Following energy minimization and thermal equilibration, an unrestrained 100-ns production MD run was conducted. Periodic boundary conditions were disabled to preserve molecular integrity, and center-of-mass translation/rotation was corrected. Root-mean-square deviation (RMSD) and fluctuation (RMSF) analyses were performed to evaluate structural stability and flexibility. Finally, the “--gmx energy” command was used to extract total energy profiles from the 100-ns trajectory.

### Construction of polycistronic expression vectors

The three consecutive tail proteins (QIG59355.1, QIG59354.1, QIG59353.1) of DSLV2 were co-expressed using a polycistronic system. Target gene sequences (GenBank: MN940570, positions 21,504–23,486) were amplified from Dishui Lake water sample genomic DNA using specifically designed primers (forward: 5′-ATGGCGACAAACTCATCATTC-3′, reverse: 5′-TTAATAATCTGCTTTTATATAGAAAGCCATC-3′) and high-fidelity PCR. Amplification products were verified by agarose gel electrophoresis for correct size before purification.

The purified fragments were cloned into the pET-30a(+) vector using *NdeI* and *XhoI* restriction sites, with QIG59353.1 engineered to contain a C-terminal His6-tag for subsequent purification. To enhance translational efficiency in *Escherichia coli*, native ribosome-binding sites between genes were replaced with *E. coli*-optimized sequences. The integrity of the polycistronic construct, including proper gene arrangement and sequence accuracy, was confirmed by Sanger sequencing prior to expression experiments.

### Expression and purification of DSLV2 three consecutive tail proteins

The polycistronic expression plasmid was transformed into chemically competent *E. coli* BL21(DE3) cells. Transformed colonies were inoculated into 200 mL of Lysogeny Broth (LB) containing 50 µg/mL kanamycin and cultured at 37°C with 200 rpm shaking until reaching mid-log phase (OD_600_ ≈ 0.8). Protein expression was induced with 0.5 mM IPTG, followed by continued incubation at 28°C for 10 h.

Cells were harvested by centrifugation (8,000 rpm, 5 min) and resuspended in 20 mL lysis buffer (50 mM Tris-HCl, pH 7.5, 300 mM NaCl, 5% glycerol, 5 mM DTT). Cell lysis was initiated by adding 200 µL of 100 mM PMSF and 100 µL of 20 mg/mL lysozyme, followed by incubation at 37°C with 150 rpm shaking for 45 min. To enhance lysis efficiency, cells underwent a freeze-thaw cycle (-80°C freezing followed by 37°C thawing). The lysate was clarified by centrifugation (12,000 rpm, 30 min, 4°C), and the soluble fraction was collected for SDS-PAGE analysis.

Protein purification was conducted using an ÄKTA pure chromatography system with a Ni-NTA affinity column. The column was pre-equilibrated with binding buffer (50 mM Tris-HCl, pH 7.5, 300 mM NaCl, 5 mM DTT) prior to sample loading. The clarified lysate was applied to the column at a flow rate of 1 mL/min, followed by extensive washing with 10 column volumes of wash buffer (identical composition to binding buffer) to remove non-specifically bound proteins. Target proteins were eluted using a linear 15–200 mM imidazole gradient in elution buffer (50 mM Tris-HCl, pH 7.5, 300 mM NaCl). Elution profiles were monitored by UV absorbance at 280 nm, and collected fractions were analyzed by SDS-PAGE to assess purity. The purest fractions were pooled and subjected to overnight dialysis at 4°C against storage buffer (50 mM Tris-HCl, pH 7.5, 300 mM NaCl, 5 mM DTT) to remove residual imidazole. The dialyzed protein solution was concentrated using 30-kDa MWCO Amicon Ultra centrifugal filters (Merck). Final protein aliquots were flash-frozen in liquid nitrogen and stored at −20°C for subsequent experiments.

### Western blotting

Protein samples were mixed with SDS-PAGE loading buffer, heat-denatured at 95°C for 5 min, and resolved by 12% SDS-PAGE. Following electrophoresis, proteins were transferred to PVDF membranes using semi-dry transfer system. The membranes were blocked with 5% non-fat milk in Tris-buffered saline with 0.1% Tween-20 (TBST) for 1 h at room temperature to prevent non-specific binding. For immunodetection, membranes were probed with mouse anti-His primary antibody (1:2,000 dilution in blocking buffer) for 1 h at room temperature with gentle agitation. After three 10-min TBST washes, membranes were incubated with HRP-conjugated goat anti-mouse secondary antibody (1:10,000 dilution) for 1 h. Protein bands were visualized using enhanced chemiluminescence substrate and detected using a ChemiDoc imaging system.

### Expression and purification of DSLV2 MCP

*In vitro* expression of the DSLV2 major capsid protein (MCP) was performed following the protocol described by Born et al. (41). The gene encoding DSLV2 MCP (QIG59351.1) was codon-optimized for *E. coli* expression and cloned into the pETM-11 vector, resulting in an N-terminal His-tagged construct. The recombinant plasmid was transformed into *E. coli* BL21 (DE3) cells. Protein expression was induced with 0.4  mM IPTG at 11°C for 36  h in the LB medium. Cells were harvested by centrifugation and lysed by sonication on ice. SDS-PAGE analysis revealed that DSLV2 MCP was expressed predominantly as inclusion bodies, which were isolated by centrifugation, washed, and solubilized in 8 M urea-containing buffer. The denatured protein was purified under denaturing conditions using Ni-NTA affinity chromatography with imidazole gradient elution. Eluted fractions containing DSLV2 MCP were pooled by stepwise dialysis into the buffer containing 50  mM Tris-HCl (pH 8.0), 200  mM NaCl, 0.5  mM EDTA, 2  mM TCEP, and 5% glycerol. The final preparation yielded ~85% pure protein at a concentration of 2  mg/mL, as determined by SDS-PAGE and BCA assay, with a total yield of approximately 18  mg. Protein aliquots were flash-frozen in liquid nitrogen and stored at −20°C for subsequent experiments.

### Negative staining electron microscopy

For sample preparation, glow-discharged Formvar/carbon-coated 100-mesh copper grids were used. A 5-µL aliquot of purified three consecutive tail proteins (0.1 mg/mL) was applied to each grid and allowed to adsorb for 2 min at room temperature. Excess sample was removed by gently blotting with a filter paper, followed by three rapid washes with distilled deionized water (ddH_2_O). Negative staining was performed by applying 0.5% (wt/v) uranyl acetate for 90 s, with excess stain removed by blotting. Samples were imaged using a Tecnai T20 transmission electron microscope (FEI/Thermo Fisher Scientific) operating at 200-kV accelerating voltage. Images were acquired at nominal magnifications ranging from 30,000× to 100,000× using a side-mounted CCD camera.

### Identification of virophage tail proteins

We retrieved all available genomes classified under the family *Lavidaviridae* (currently reclassified as *Virophaviricetes*) from the NCBI nucleotide database (accessed August 2023) (2, 17). From the IMG/VR v4 high-confidence database, we selected sequences >10 kb that were classified as *Lavidaviridae* (42). Initial screening using taxonomic classification software (2) identified 2,517 confirmed virophage sequences.

Open reading frame (ORF) prediction was performed using Geneious Prime with stringent parameters (ATG start codon, minimum length = 150 bp). Predicted proteins were analyzed by BLASTp against the NCBI nr virus database (E-value cutoff: 1e-5). Candidate tail proteins were identified through sequence similarity to DSLV2 tail proteins or other known tail-associated proteins, followed by validation using HHPred (43) against multiple databases (Pfam-A_v35, PDB_mmCIF70, COG_KOG_v1.0, PGROGs_v4). This comprehensive analysis identified 285 tail protein-encoding genes across 169 distinct virophages.

### Synteny analysis

Genomic regions encoding the three consecutive tail proteins were analyzed using Clinker (https://cagecat.bioinformatics.nl/tools/clinker), a bioinformatics tool for comparative genomics visualization (44). The analysis was performed with a stringent alignment similarity threshold of 30% to ensure that only biologically meaningful sequence conservation was retained.

### Phylogenetic analysis

Two distinct phylogenetic trees were reconstructed: (i) a MCP tree incorporating MCP sequences from all 45 virophages containing three consecutive tail proteins and representative members from each of the seven virophage families; and (ii) a tail protein phylogeny based on complete sets of three consecutive tail protein sequences and their top BLASTP matches (E-value <10^−5^) from the NCBI RefSeq database. All significant matches were bacteriophage tail proteins with redundancy reduced using CD-HIT (90% sequence identity threshold) (45). Additionally, the DSLV2 tail proteins were analyzed separately against all these BLASTP top hits to examine their specific evolutionary relationships.

Multiple sequence alignments for all phylogenetic analyses were generated using MAFFT v7.450 with default parameters, followed by trimming with trimAL v1.2 with the parameters “-automated1” to remove poorly aligned regions (46, 47). Maximum-likelihood trees were constructed from the trimmed alignments using FastTree v2.1.11 under the WAG substitution model with gamma-distributed rate heterogeneity and visualized using ChiPlot (https://www.chiplot.online/) (48).

### Molecular docking simulations with potential receptors

Phylogenetic reconstruction (see Results below) revealed that the three consecutive tail proteins of DSLV2 clustered closely with the tail fiber protein (YP_009322593.1) from *Synechococcus* phage SAM7 of *Synechococcus* WH7803 (49). This evolutionary relationship prompted us to investigate potential interactions between these viral proteins and their putative receptors in *Synechococcus* WH7803. Consistent with established mechanisms of phage-host interaction (50), we selected two classes of Gram-negative bacterial surface molecules as potential receptors for our docking studies: outer membrane porins (OMPs: UniProt A5GKF4, A5GKY9, A5GM90) and LPS components. Using AlphaFold3 (36), we predicted interaction interfaces between viral tail proteins (modeled as both hetero- and homotrimers) and OMP trimers. The minimal core LPS structure of *Synechococcus* WH7803 was reconstructed based on its annotated biosynthesis pathway in KEGG (51). Molecular interactions between tail protein oligomers and the modeled LPS were simulated using AutoDock Vina with default parameters (52).

### Binding assay

The binding of *Synechococcus* cells to the DSLV2 tail protein heterotrimer (His-tagged) was evaluated using an enzyme-linked immunosorbent assay (ELISA). *Synechococcus elongatus* and *Synechococcus* sp. strains were obtained from the Freshwater Algae Culture Collection at the Institute of Hydrobiology, Wuhan, China. *E. coli* BL21, used as a negative bacterial control strain, was sourced from laboratory stocks. *Synechococcus* cultures were grown in BG11 liquid medium at 37°C, and *E. coli* BL21 was cultured in the LB medium at 37°C. Cells were harvested by centrifugation, washed three times with phosphate-buffered saline (PBS) to remove residual medium, and resuspended to a final concentration of 10⁶ cells/mL for all subsequent steps. For ELISA, 50  µL of carbonate coating buffer and 50  µL of cell suspension were added per well in a 96-well ELISA plate (in triplicate for each strain), followed by incubation at 37°C for 2  h. Wells were then washed with PBS containing 0.05% Tween-20 (PBS-T) and blocked with 200  µL of blocking buffer at 4°C overnight. For the cell-free negative control, PBS was added in place of cell suspension prior to blocking. After washing, 100  µL of DSLV2 tail protein heterotrimer (0.5  µg) was added to each well and incubated at 4°C overnight. For the tail protein-free control, 100  µL of PBS was added instead of protein. Wells were then washed and incubated with rabbit anti-His primary antibody (1:1,000 dilution) at 37°C for 2  h, followed by HRP-conjugated anti-His secondary antibody (1:3000 dilution) at 37°C for 1  h. After final washes, TMB substrate was added for color development, and absorbance at 450  nm was measured using a microplate reader. For the LPS antibody competition assay, cells were first incubated with general anti-LPS antibody (catalog no. MK52671S, Abmart, China) prior to the addition of tail protein using the same protocol. For the MCP binding assay, the procedure was identical to that of the tail protein assay, with MCP protein substituted in place of DSLV2 tail protein. Data were analyzed using GraphPad Prism with one-way ANOVA to assess statistical significance.

## RESULTS

### Structural similarity between DSLV2 and T4 phage tail proteins

**Fig 1:**
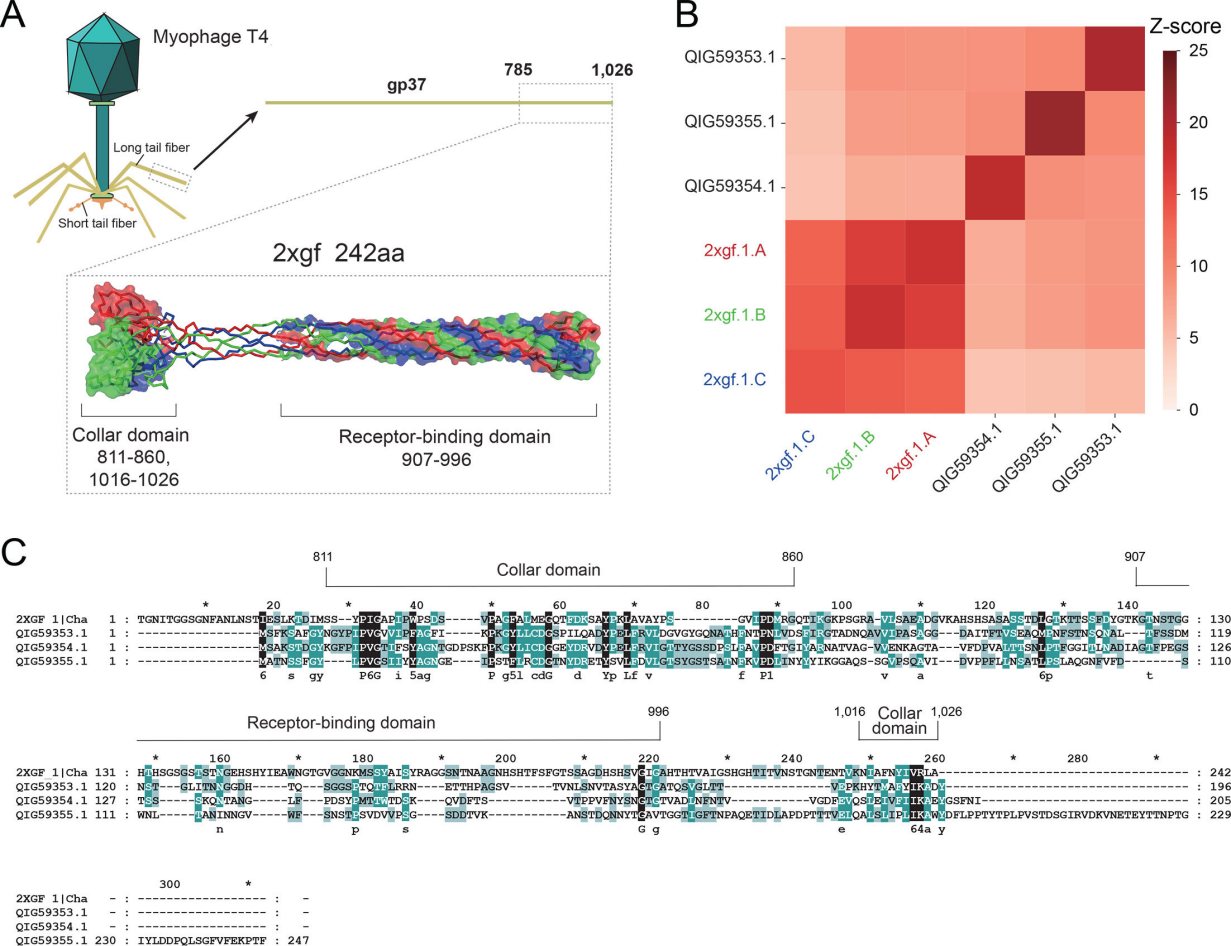
Structural and sequence comparison of DSLV2 tail proteins and bacteriophage T4 long tail fiber tip. (**A**) Architecture of bacteriophage T4 long tail fiber and its receptor-binding tip. Chains A (red), B (green), and C (blue) of the gp37 tip (residues 785–1,026; PDB: 2XGF) are shown, with identified collar (PF07484) and receptor-binding domains. (**B**) Hierarchical clustering dendrogram based on DALI-derived pairwise Z-scores, demonstrating structural relationships among homologous proteins. (**C**) Multiple sequence alignment of DSLV2 tail proteins and T4 gp37 tip (residues 785–1,026). Conservation levels: identical (black), highly similar (dark cyan), and moderately similar (light cyan) residues. Structural/functional landmarks are denoted by asterisks (*) for decile positions, lowercase labels for key residues, and bracketed numbers marking domain boundaries (collar and receptor-binding domains).

To investigate the functional potential of the three consecutive tail proteins (QIG59353.1, QIG59354.1, QIG59355.1) (196 aa, 205 aa, and 247 aa) in DSLV2, we performed structural homology searches using DALI. All three proteins returned the bacteriophage T4 needle-shaped, receptor-binding long tail fiber tip (gp37, residues 785–1,026; PDB ID: 2XGF) as their top structural homologs (Fig. 1A), with Z-scores >6.5, confirming significant structural similarity (Fig. 1B).

Meanwhile, sequence alignment analysis demonstrated that the DSLV2 tail proteins exhibit sequence similarity (15.1%–17.7%) to the collar domain (residues 811–860 and 1,016–1,026) of the gp37 (residues 785–1,026), but significantly lower conservation (7.3%–15.9%) with the receptor-binding domain (residues 907–996) (Fig. 1C). This domain organization was further supported by Conserved Domain Database searches, which confirmed the presence of a conserved collar domain (likely critical for stability). Although no conserved domain was detected within the putative receptor-binding region, its lower sequence conservation suggests that it may represent a variable region potentially involved in host-specific interactions.

### Three consecutive tail proteins of DSLV2 form a heterotrimeric structure

The three consecutive tail proteins of DSLV2 share comparable length and structural features with the gp37 tip region (residues 785–1,026), which is known to form a stable homotrimer (38) (Fig. 1A). Based on these similarities, we propose two potential assembly models for the DSLV2 tail proteins: (i) three distinct homotrimers or (ii) a single heterotrimeric complex.

**Fig 2:**
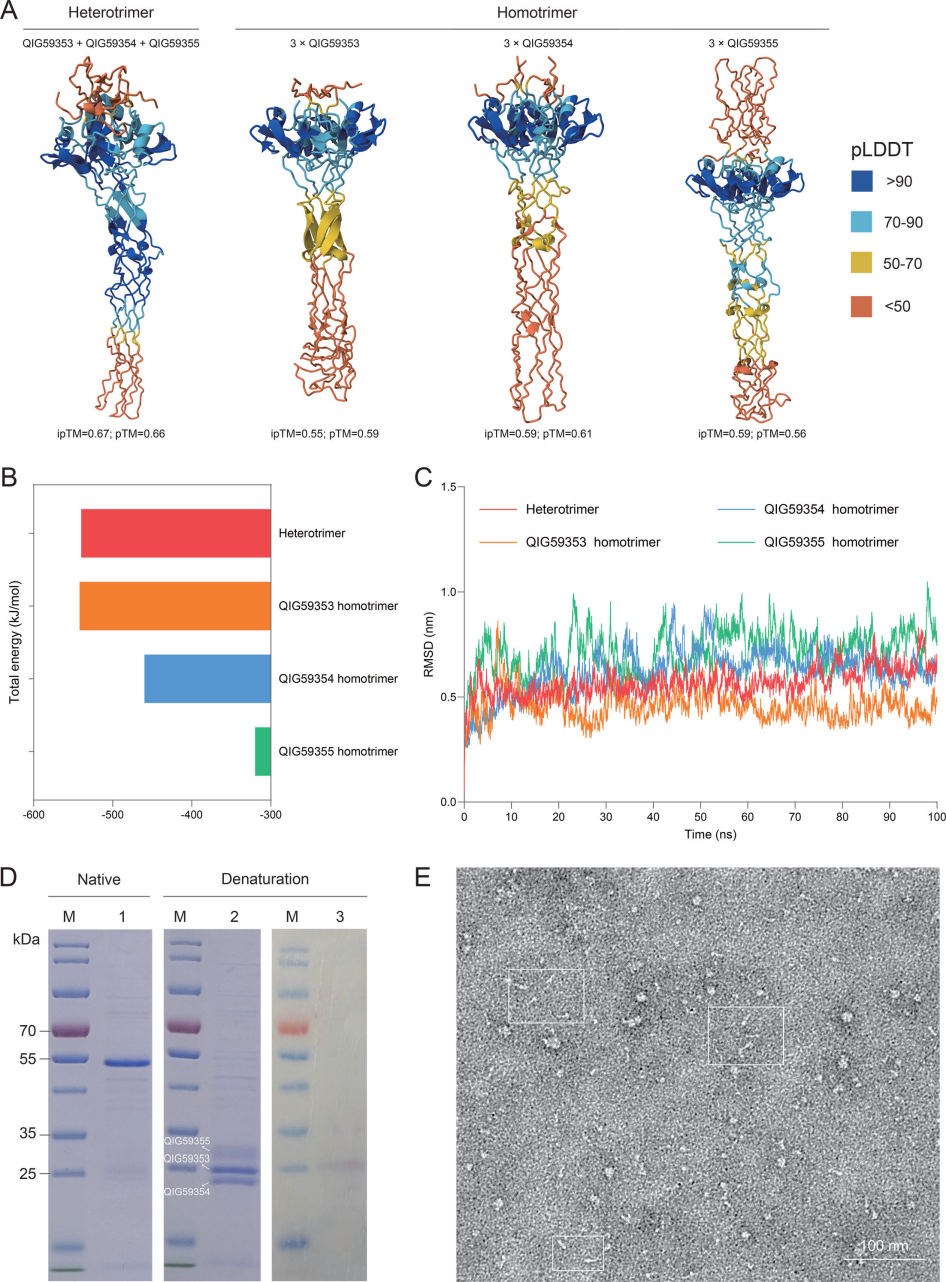
Structural and biochemical characterization of DSLV2 tail protein heterotrimer. (**A**) Predicted trimeric structures of DSLV2 tail proteins. Left: heterotrimeric assembly of three consecutive tail proteins. Middle-left to right: homotrimeric structures formed by individual proteins (QIG59353.1, QIG59354.1, and QIG59355.1). Structures are colored by pLDDT confidence scores: dark blue (>90, high), light blue (70–90, good), yellow (50–70, moderate), and orange (<50, low). (**B**) Potential energy profile of the trimeric complex during 100-ns MD simulation, where more negative values indicate enhanced stability. (**C**) RMSD trajectory of the trimer during simulation, with smaller fluctuations reflecting structural stability. (**D**) Electrophoretic analysis of co-expressed proteins. M: protein marker; Lane 1: native PAGE; Lanes 2–3: SDS-PAGE (Lane 3: Western blot). In Lane 2, bands correspond to QIG59355 (top), QIG59353 (middle), and QIG59354 (bottom). (**E**) Negative-stain EM of purified protein complex (scale bar: 100 nm). A white square highlights the representative fibrous structures (~16 nm length).

To resolve this structural ambiguity, we employed AlphaFold3 for complex prediction. The modeling results demonstrated that the heterotrimeric complex achieved significantly higher confidence scores (ipTM = 0.67, pTM = 0.66) compared to homotrimeric alternatives (Fig. 2A) and that the predicted heterotrimeric structure maintains domain architecture analogous to gp37 (Fig. 1A).

To assess complex stability, we performed 100 ns MD simulations. Heterotrimer and QIG59353.1 homotrimer showed favorable energy landscapes, while QIG59354.1 and QIG59355.1 homotrimers exhibited less stable energy profiles (Fig. 2B). Moreover, the heterotrimer displayed minimal fluctuations (RMSD range: 5.7 ± 2.6 Å). In contrast, QIG59353.1 homotrimer showed moderate stability (RMSD range: 4.6 ± 4 Å), and other homotrimers (RMSD range: 6.2 ± 3.5 Å/7.4 ± 5.9 Å) demonstrated significant structural drift (Fig. 2C).

To biochemically characterize the predicted heterotrimeric complex, we employed a polycistronic expression system in *E. coli* co-expressing all three DSLV2 tail proteins (QIG59353.1-His, QIG59354.1, and QIG59355.1). Native PAGE revealed a dominant band at ~55 kDa (Fig. 2D, Lane 1), consistent with the expected mass of a heterotrimeric complex (theoretical mass: 58.3 kDa). SDS-PAGE resolved three distinct bands corresponding to each subunit (Fig. 2D, Lane 2). Western blot confirmed selective detection of His-tagged QIG59353.1 (Fig. 2D, Lane 3). These results demonstrate that QIG59354.1 and QIG59355.1 co-purify specifically with His-tagged QIG59353.1, the subunits maintain stable interactions under native conditions, and the complex dissociates only upon denaturation.

Moreover, negative-staining EM visualized abundant fibrous structures (~16 nm length) (Fig. 2E). The dimensions and morphology closely match the predicted heterotrimeric model and the characteristic needle-like architecture of RBPs (Fig. 1A and 2A).

Collectively, the three tail proteins form a specific, stable heterotrimeric complex; the observed 1:1:1 stoichiometry supports the computational predictions; and the elongated structure suggests functional conservation with phage RBPs while maintaining virophage-specific features.

### Receptor-binding region of DSLV2 tail protein heterotrimer

**Fig 3:**
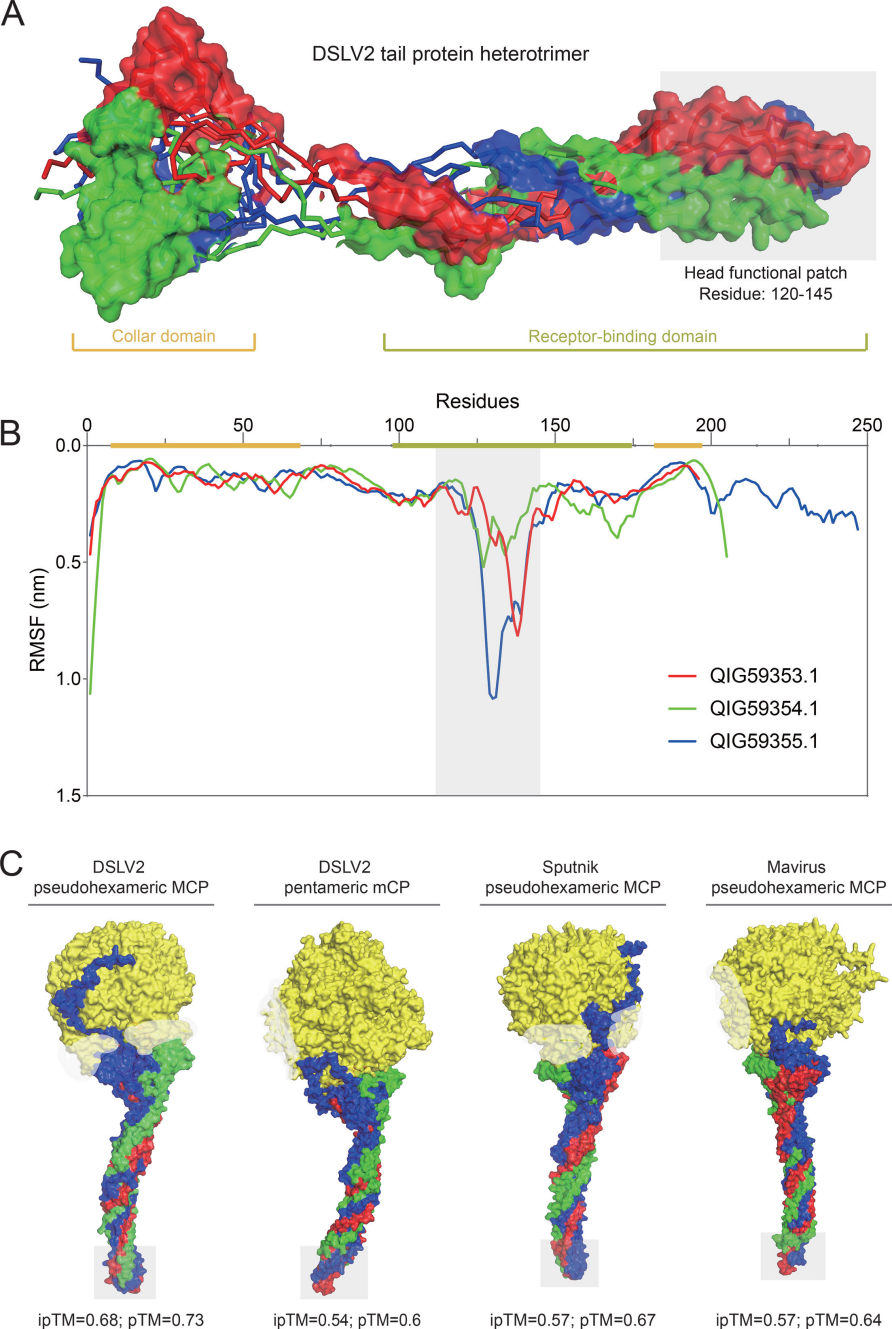
Receptor-binding region of the DSLV2 tail protein heterotrimer. (**A**) Predicted heterotrimeric structure with chains QIG59353.1 (red), QIG59354.1 (green), and QIG59355.1 (blue). The collar domain and receptor-binding domain are indicated. Head functional patch is shaded gray. (**B**) RMSF profile from 100-ns MD simulation. Color-coded residue ranges (*x*-axis) correspond to domains in panel **A**. The gray-shaded region (residues 120–145) marks a conformationally flexible segment (head functional patch in panel **A**). (**C**) Predicted binding interfaces between the DSLV2 heterotrimer (chains colored as in panel** A**) and viral capsomers: DSLV2 pseudohexameric MCP (left), DSLV2 pentameric minor capsid protein (mCP; middle-left), Sputnik MCP (middle-right), and Mavirus MCP (right). Capsomer proteins (yellow) are shown with semi-transparent capsid shell (white). Head functional patch, marked in panels **A and B**, is shaded gray.

Through integrated sequence-structure analysis, we identified both the collar domain and receptor-binding domain within the heterotrimeric complex (Fig. 3A), revealing an architectural organization analogous to phage T4 gp37 (residues 785–1,026; Fig. 1A). MD simulations demonstrated pronounced dynamic behavior in the head functional patch (residues 120–145; Fig. 3A and B), characterized by (i) substantially elevated flexibility (RMSF = 7.5 Å versus 1.8 Å in the collar domain), (ii) distinctive loop mobility profiles, and (iii) residual fluctuation patterns consistent with binding-competent conformations. These combined structural and dynamic attributes provide compelling evidence for the head functional patch’s involvement in receptor recognition and binding.

### Fibrous tail proteins may protrude from pseudohexameric capsomer

Structural predictions using AlphaFold3 revealed detailed interactions between the DSLV2 tail protein heterotrimer and its associated pseudohexameric/pentameric capsomers (Fig. 3C). Comparative analyses with Sputnik and Mavirus pseudohexameric capsomers served as validation controls (Fig. 3C). The modeling demonstrated high-confidence binding (ipTM = 0.68, pTM = 0.73) between the heterotrimer’s collar domain and the outer surface of the major capsid pseudohexamer (31). These results strongly suggest that the fibrous tail assembly, comprising three consecutive tail proteins, extends outward from the pseudohexameric capsomer on the DSLV2 capsid surface. This structural arrangement likely contributes to virion stability while positioning the exposed head region for potential receptor recognition.

### Three consecutive tail proteins widely present in Aquatic Virophage 1 lineage

**Fig 4:**
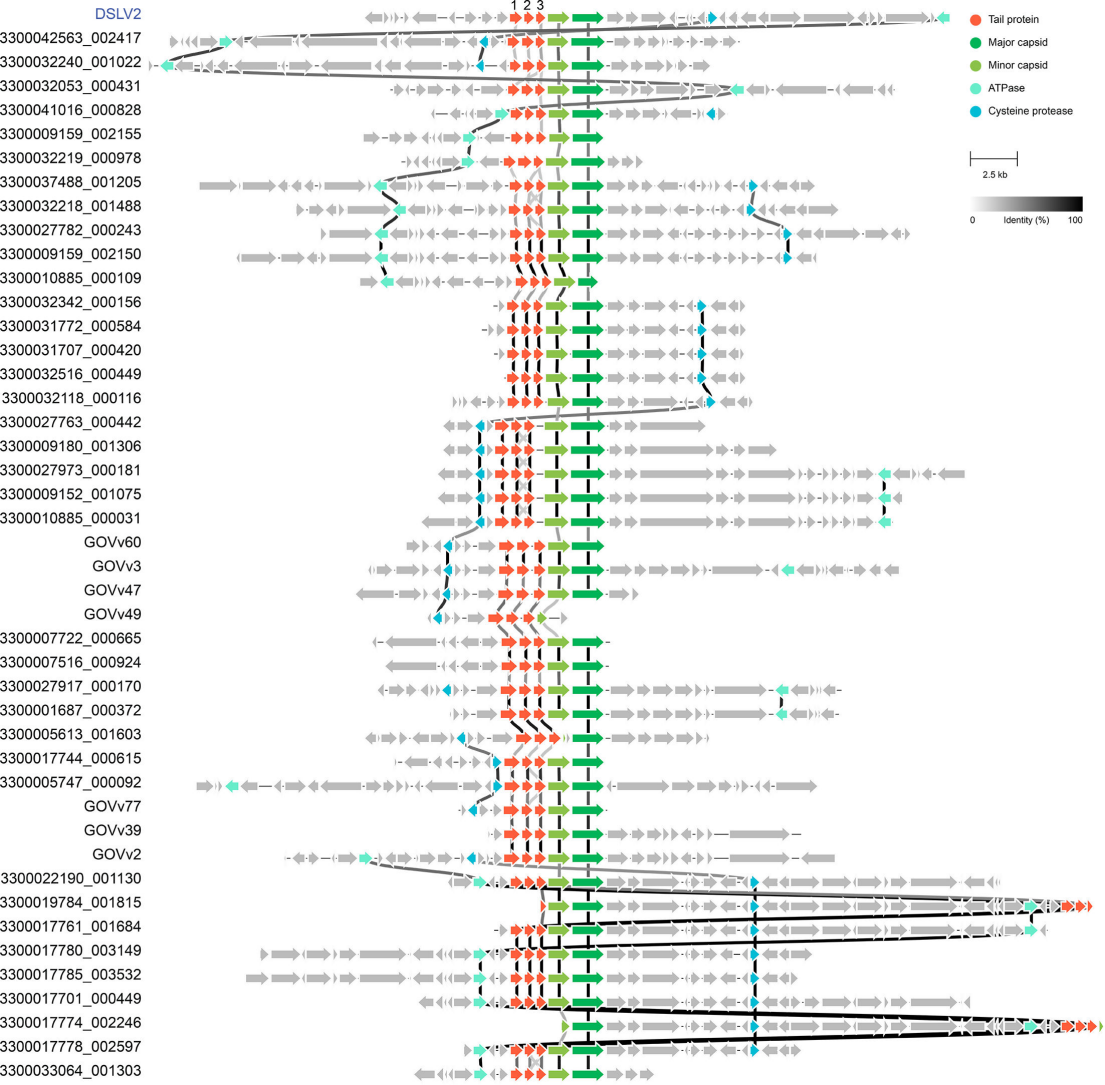
Genomic synteny analysis of 45 virophages containing three consecutive tail protein genes. ORFs are represented by arrows, with color coding indicating functional categories: four conserved core virophage genes and three consecutive tail protein genes. All displayed protein homologs share >30% amino acid identity. Linkage colors between orthologous genes are weighted by sequence similarity, with darker hues representing higher identity levels.

To investigate the prevalence of the tripartite tail fiber gene clusters among virophages, we performed a systematic genomic survey of all currently available virophage sequences (*n* = 3,029). Remarkably, we identified 44 virophages (1.5% of total) that maintain this distinctive three-gene architecture, and these virophages exhibit striking genomic conservation with DSLV2 (Fig. 4). All three tail protein genes share identical transcriptional orientation, with the 3′-terminal gene invariably positioned adjacent to the minor capsid protein gene (Fig. 4). This syntenic conservation across diverse virophages suggests strong evolutionary maintenance, potentially driven by functional constraints or requirements for coordinated expression. Domain analysis revealed that all three proteins contain the phage tail collar domain (PF07484) and are consistently annotated as tail fiber components (https://figshare.com/s/7af50e7d73292a835549), confirming their structural and functional relatedness.

**Fig 5:**
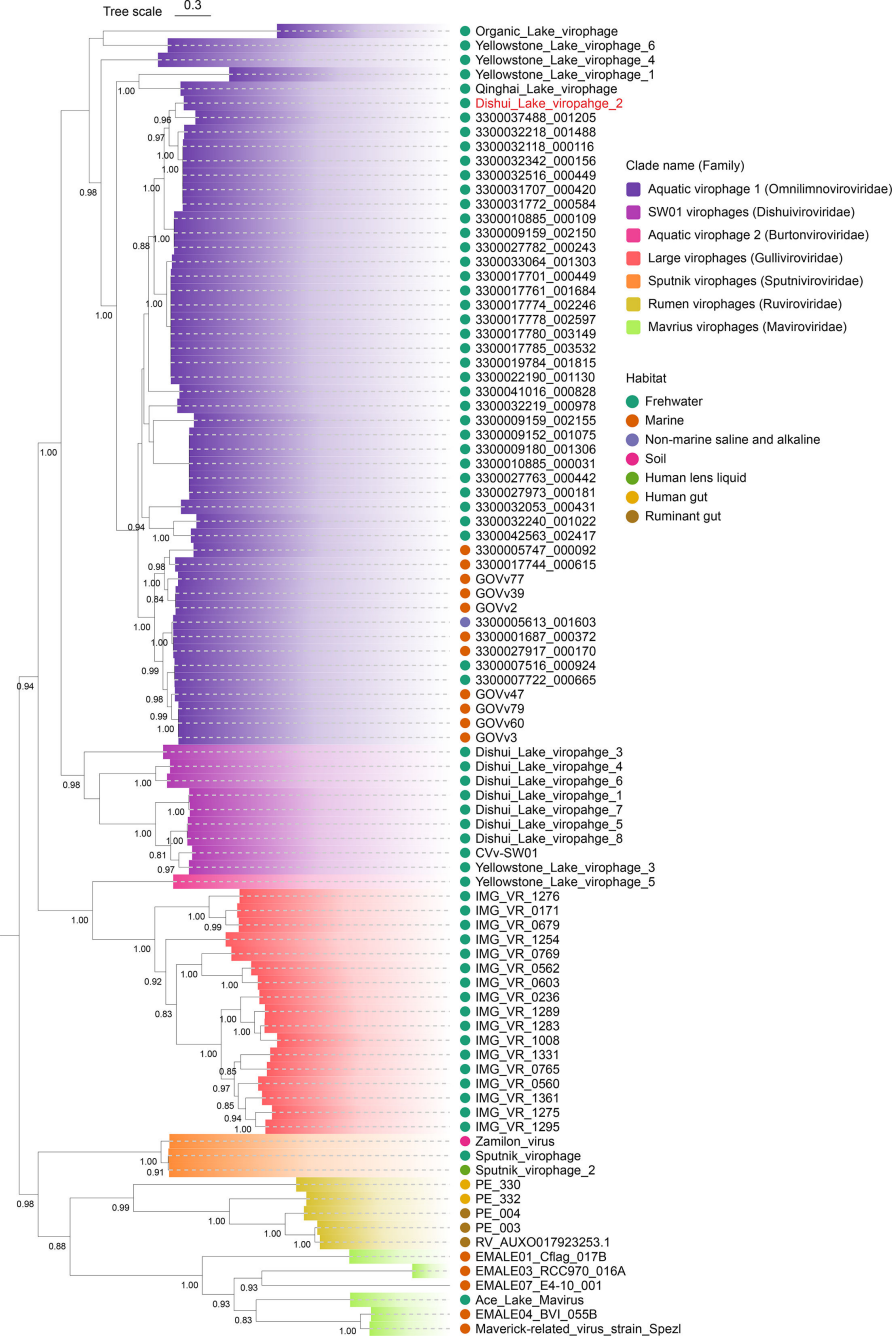
Maximum-likelihood phylogenetic tree of MCPs from virophages containing three consecutive tail proteins and reference virophages. Colored blocks (branch tips to nodes) represent distinct virophage families. Habitat origins are indicated by colored dots.

Phylogenetic analysis of virophage capsid proteins revealed that all 44 identified virophages, along with DSLV2, form a monophyletic clade within Aquatic Virophage 1 lineage (*Priklausovirales; Omnilimnoviroviridae*) (Fig. 5). This strongly supports their shared evolutionary origin and suggests conserved biological functions, particularly in algae-associated CVv systems. While all members possess the characteristic tripartite tail proteins, freshwater-originating virophages (including DSLV2) form a distinct subclade from their marine counterparts (Fig. 5). This phylogenetic separation correlates with habitat differences (freshwater versus marine ecosystems), implying potential adaptive divergence in tail protein function.

### Evolutionary conservation and divergence of tripartite tail proteins in virophages

To elucidate the evolutionary dynamics of the three consecutive tail proteins, we performed phylogenetic analysis of their amino acid sequences, using the top BLASTp hit from the NCBI reference database as representative sequences for each protein. Meanwhile, based on their genomic positions in the tripartite array (first, second, and third—the latter adjacent to the minor capsid protein; Fig. 4), we classified the proteins and reconstructed their phylogenetic relationships.

**Fig 6:**
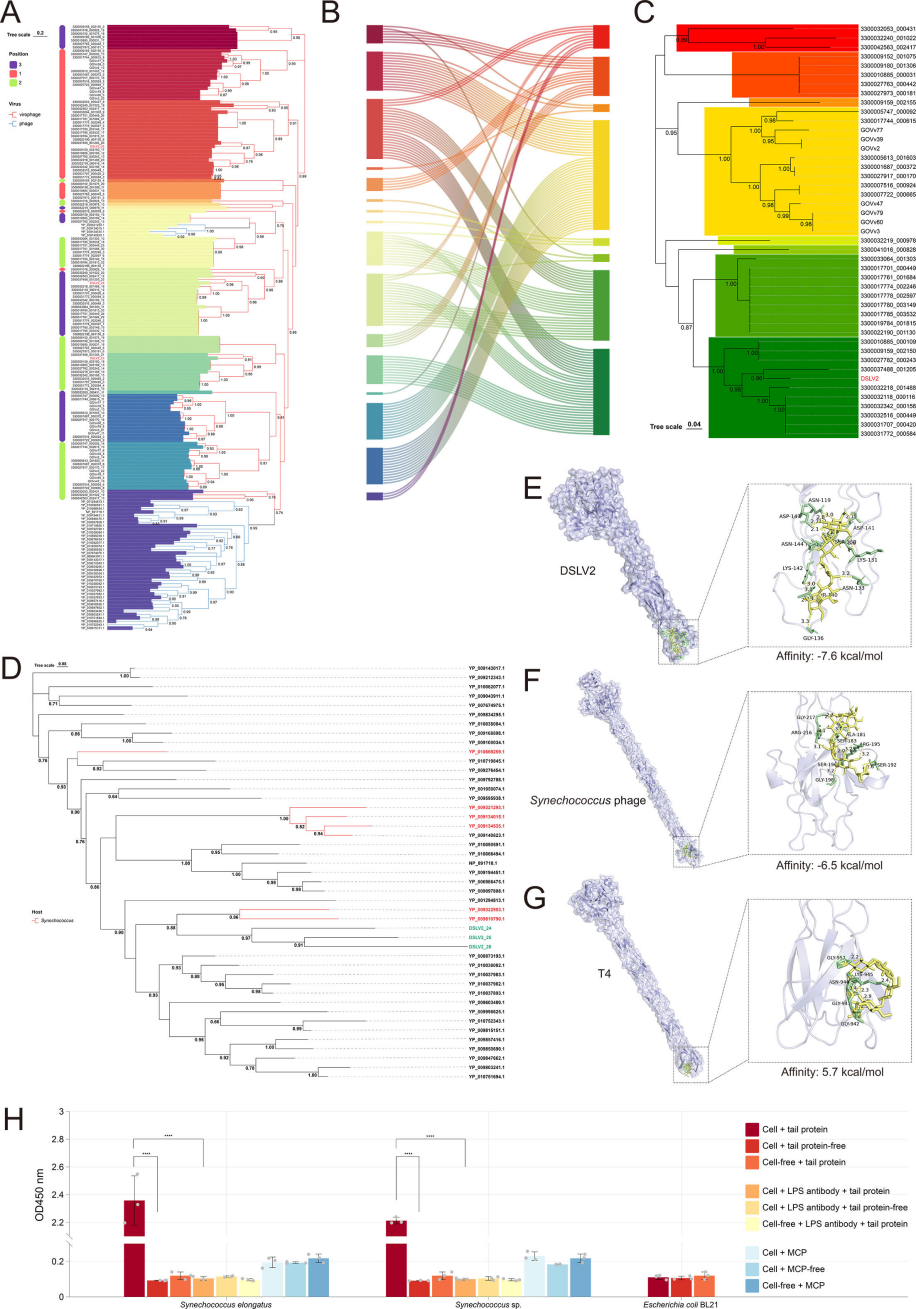
Phylogenetic relationships and receptor-binding architectures of virophage tail proteins. (**A**) Maximum-likelihood phylogenetic tree of tail protein sequences. Branch colors denote viral origin (phage: blue; virophage: orange). Colored blocks (branch tips to nodes) represent well-supported clades (bootstrap >80). Left-side labels indicate positional conservation: "1" (QIG59353.1), "2" (QIG59354.1), and "3" (QIG59355.1; adjacent to mCP gene) in the tripartite array. (**B**) Sankey diagram mapping evolutionary relationships between tail proteins (left) and their cognate MCPs (right). Color-matched blocks represent corresponding clades between phylogenies. (**C**) Virophage MCP phylogeny (colored blocks as in panel **A**). (**D**) Phylogenetic analysis of DSLV2 tripartite tail proteins (colored in cyan) and phage homologs (*Synechococcus* phage tail proteins colored in red). (**E–G**) Predicted binding interfaces between (**E**) DSLV2 heterotrimer, (**F**) *Synechococcus* phage homotrimer, and (**G**) T4 phage homotrimer, with *Synechococcus* minimal core LPS (colored in yellow and green; enlarged insets). (**H**) Binding of DSLV2 heterotrimeric tail proteins to *Synechococcus* cells. The data were collected from three independent replicates. ****P < 0.0001 (one-way ANOVA).

The phylogenetic tree (Fig. 6A) revealed distinct clades corresponding to each tail protein position (first, second, third). Proteins occupying the same position exhibited higher sequence similarity to one another than to their counterparts within the same virophage, indicating strong positional conservation across diverse virophages. However, sequence divergence was evident within each positional group, as reflected by their distribution across multiple phylogenetic branches.

By integrating a Sankey diagram (Fig. 6B) to map tail protein phylogeny (Fig. 6A) onto virophage MCP evolution (Fig. 6C), we observed that tail proteins from the same positional group but different phylogenetic branches were consistently associated with MCPs from distinct clades. This parallel diversification suggests that tail proteins and their cognate virophages may have undergone coevolution, with positional specificity maintained despite sequence divergence.

Collectively, the three tail proteins demonstrate dual evolutionary signatures—strict conservation of their relative genomic positions coupled with sequence-level diversification. This pattern highlights a fundamental architectural principle in virophages harboring tripartite tail proteins, where positional fidelity is preserved even as the proteins themselves evolve along virophage-specific trajectories. Such features likely reflect functional constraints on tail assembly while allowing adaptive flexibility in receptor interactions.

### Three consecutive tail proteins in DSLV2 are evolutionarily related to those of a *Synechococcus* phage

Our phylogenetic reconstruction uncovers that bacteriophage tail proteins formed two distinct monophyletic groups, each of which robustly clustered (bootstrap >0.85) with separate virophage tail protein lineages (Fig. 6A). This topology supports multiple independent horizontal gene transfer (HGT) events between bacteriophages and virophages.

Furthermore, the three DSLV2 tail proteins phylogenetically formed a distinct, well-supported clade (bootstrap value = 0.876) with tail proteins from *Synechococcus* phages in the reconstructed evolutionary tree (Fig. 6D). This strong phylogenetic affinity suggests a possible HGT event between *Synechococcus* phages and DSLV2.

### Potential binding receptor of DSLV2 heterotrimeric tail proteins

**Fig 7:**
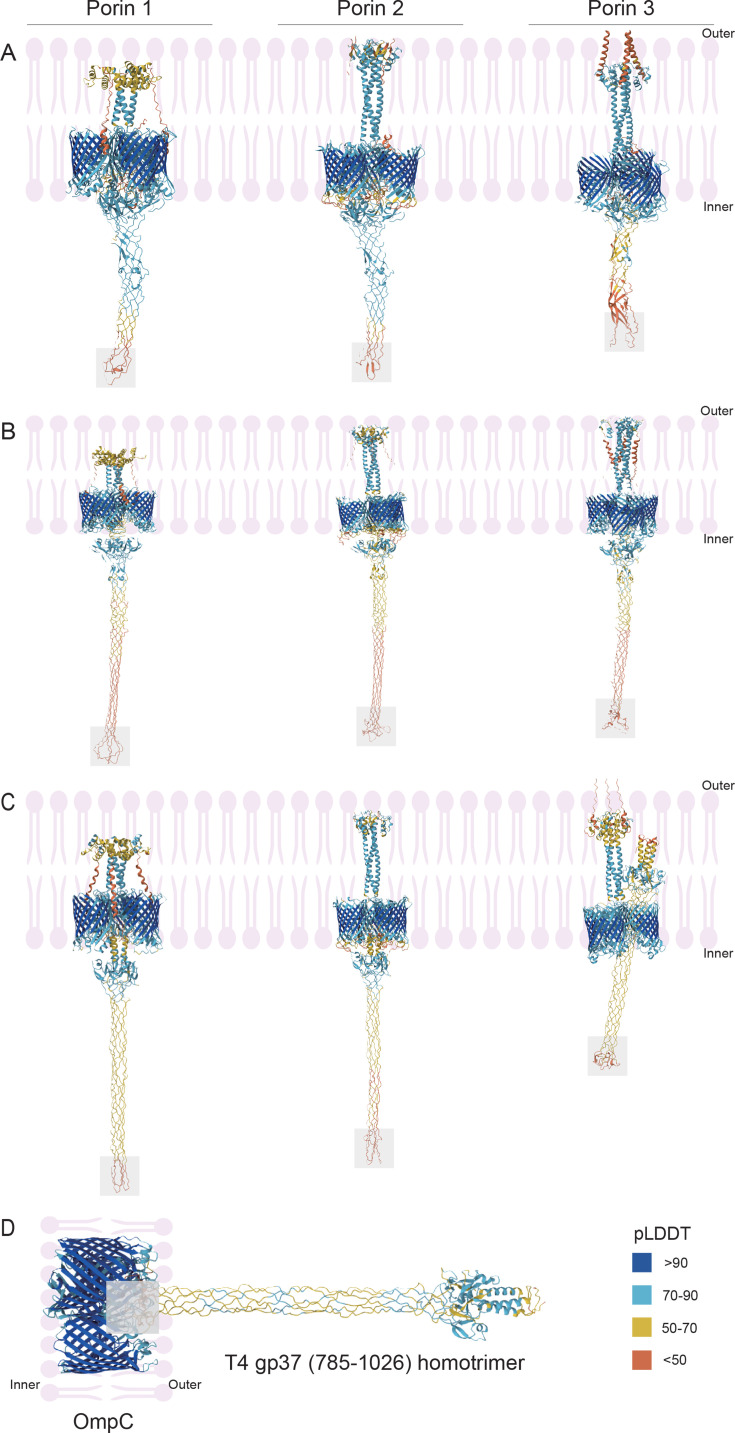
Predicted interaction interfaces between viral tail trimers and bacterial OMPs. (**A–C**) Structural models of protein-polymer complexes between (**A**) DSLV2 heterotrimeric tail proteins, (**B**) *Synechococcus* phage SCAM7 homotrimeric tail proteins, and (**C**) T4 phage homotrimeric tail proteins (gp37, residues 785–1,026), with *Synechococcus* WH7803 porin trimers (Porin 1/2/3; with the phospholipid bilayer prominently highlighted in pink to indicate the outer membrane environment). Each panel shows three binding conformations (left to right) representing distinct porin variants. The functional patch on the viral tail trimer head is shaded gray. (**D**) Positive control: T4 gp37 homotrimer bound to *E. coli* OmpC porin (PDB: 2J1N), validating the prediction method.

The close evolutionary relationship between DSLV2 tail proteins and *Synechococcus* phage tail fibers suggests conserved receptor recognition mechanisms (Fig. 6D). As *Synechococcus* (a Gram-negative cyanobacterium) typically interacts with phage proteins targeting LPS or OMPs (50), we hypothesized similar recognition patterns for DSLV2. However, neither DSLV2 heterotrimeric tail proteins nor *Synechococcus* phage homotrimeric fibers showed binding to porins, while positive control with T4 phage tail fibers successfully reproduced known porin interactions (validating our computational approach) (Fig. 7). By contrast, LPS binding specificity revealed distinct interaction profiles: −7.6 kcal/mol (strongest LPS affinity) for DSLV2 heterotrimer, −−6.5 kcal/mol for *Synechococcus* phage fiber (positive control), and −5.7 kcal/mol (weakest) for T4 phage tail fiber (negative control) (Fig. 6E through G). The superior LPS binding affinity of DSLV2 tail proteins (Fig. 6E) suggests that LPS may serve as the primary receptor for DSLV2 adsorption. Accordingly, these findings support a model where DSLV2 has evolved to recognize *Synechococcus* surface LPS, potentially facilitating its ecological persistence in cyanobacteria-rich aquatic environments.

### *In vitro* binding between *Synechococcus* and DSLV2 heterotrimeric tail proteins

To experimentally investigate the binding capability of DSLV2 heterotrimeric tail proteins, we performed ELISA using *Synechococcus* cells (two species) as targets. A pronounced binding signal was observed in wells coated with *Synechococcus* cells and incubated with the DSLV2 tail protein, with an average absorbance of A450 = 2.36  ±  0.15 and A450 = 2.21  ±  0.02, significantly higher than in all control groups (*P* < 0.0001, one-way ANOVA) (Fig. 6H). In contrast, wells with *Synechococcus* cells but without the tail protein, or those with tail protein but no cells, showed negligible signals (A450 = 0.09–0.12), confirming assay specificity (Fig. 6H). Importantly, preincubation of cells with anti-LPS antibody resulted in a drastic reduction of binding signal (A450 = 0.10–0.11), indicating that LPS structures on the *Synechococcus* surface are involved in tail protein recognition (Fig. 6H). Substitution of the tail protein with the unrelated MCP of DSLV2 yielded no significant binding (A450 = 0.18–0.23), further supporting the specificity of the interaction (Fig. 6H). In contrast, the negative control bacterium *E. coli* BL21 showed no detectable binding under any tested condition (A450 = 0.10–0.12), reinforcing the selective affinity of DSLV2 tail proteins for *Synechococcus* (Fig. 6H). These results collectively indicate that the DSLV2 tail heterotrimers specifically bind to surface-exposed LPS moieties of *Synechococcus*, implicating these glycoconjugates as putative viral receptors during none-host recognition.

## DISCUSSION

Fiber-like appendages represent a frequently observed structural feature among viruses possessing double jelly-roll capsid architectures (53–56). These filamentous projections serve dual functional roles: (i) providing structural reinforcement by stabilizing individual capsomer interactions within the viral capsid and (ii) facilitating host recognition through RBP domains located at their distal ends (29). As molecular mediators of viral attachment, fiber RBPs execute critical functions in the infection cycle by specifically recognizing host cell surface receptors, initiating conformational changes required for viral entry, and transducing signals for subsequent infection steps (50, 57, 58).

Here, our integrated analyses reveal striking parallels between DSLV2 tail proteins and bacteriophage recognition systems. Comparative modeling reveals that the DSLV2 tripartite tail proteins exhibit significant structural similarity (Z-scores >6.5) with the gp37 tail fiber (residues 785–1,026) of bacteriophage T4 (Fig. 1A). Despite low sequence identity, these proteins retain a conserved collar domain architecture (PF07484) that maintains structural integrity, as well as a receptor-binding domain topology. Moreover, MD simulations (100-ns trajectories) reveal spontaneous formation of stable heterotrimers (ΔG = −540 kcal/mol) and head region flexibility (RMSF = 7.5 Å) essential for conformational adaptation during receptor engagement (Fig. 3B). The structural convergence suggests preservation of trimeric organization for mechanical advantage and adaptation of structurally analogous motifs for virophage-specific needs.

Importantly, the presence of three consecutively arranged tail protein genes is not exclusive to DSLV2 but represents a widely conserved genomic feature among virophages within the Aquatic Virophage 1 lineage (Fig. 5). This syntenic gene cluster, invariably positioned adjacent to the minor capsid protein gene, suggests a co-regulated functional module that may be optimized for capsid assembly or receptor interaction. Its preservation across both freshwater and marine virophages underscores its ecological success in diverse aquatic ecosystems. Notably, despite this conserved genomic architecture, sequence divergence among lineages implies that these tail proteins have undergone adaptive specialization, potentially reflecting distinct receptor-specific interactions or environmental constraints.

Phylogenetic analysis of the three tail proteins revealed an intriguing evolutionary pattern: rather than clustering by species, proteins occupying the same relative genomic position consistently grouped together across different virophages (Fig. 6A). This positional phylogenetic conservation suggests that each protein within the heterotrimeric complex maintains a distinct functional role, subject to independent evolutionary pressures. Such modular evolution points toward a division of labor within the tail apparatus, where individual subunits may contribute uniquely to receptor binding or structural stability. These findings challenge the conventional view of tail proteins evolving strictly as a single functional unit (32, 38, 50, 59), instead supporting a model of fine-tuned structural specialization shaped by virophage-receptor coevolution.

Collectively, these observations suggest that Aquatic Virophage 1 members have evolved a sophisticated strategy: maintaining a tripartite tail system that balances structural conservation with sequence-level adaptability. This modular architecture may enable virophages to retain core functional integrity while acquiring the flexibility to engage with a broad range of receptors—a critical advantage in dynamic aquatic environments.

Critically, our findings suggest that the tripartite tail protein cluster in DSLV2 likely reflects historical gene sharing with *Synechococcus* phages. Phylogenetic reconstruction reveals that DSLV2’s tail proteins form a monophyletic clade with those of *Synechococcus* phages (Fig. 6D), exhibiting a high degree of sequence homology and evolutionary relatedness. This provides compelling evidence for genetic exchange between virophages and bacteriophages, though it could also result from structural convergence driven by analogous selective pressures on receptor-binding architectures during recognition or infection processes. We propose that algae-associated virophages like DSLV2 may have co-opted phage-like tail structures not for canonical host infection but rather for non-host attachment—potentially facilitating environmental persistence or alternative interaction mechanisms. Such adaptive repurposing of bacteriophage-like modules underscores the evolutionary plasticity of virophages and highlights their capacity to exploit genetic material across disparate viral groups.

Adherence to non-host surfaces represents a notable and relatively common viral survival strategy that enhances environmental persistence while increasing the likelihood of host contact. A well-documented example is poliovirus, which binds to bacterial components such as LPS and peptidoglycan, thereby improving both virion stability and receptor-mediated entry into host cells (60). Similarly, human noroviruses interact with commensal gut bacteria, which express histo-blood group antigen-like molecules on their surfaces. These interactions not only stabilize viral particles but also facilitate infection of susceptible B cells (61). Beyond host-associated transmission, environmental bacteria can sequester noroviruses in aquatic ecosystems, promoting their accumulation in oysters through filter-feeding on virus-bound bacteria—a key mechanism in foodborne outbreaks (62). Given these precedents, the observed association between DSLV2 and *Synechococcus* may reflect an analogous ecological adaptation, wherein the virophage exploits phage-like tail structures for environmental hitchhiking, thereby optimizing transmission efficiency.

**Fig 8:**
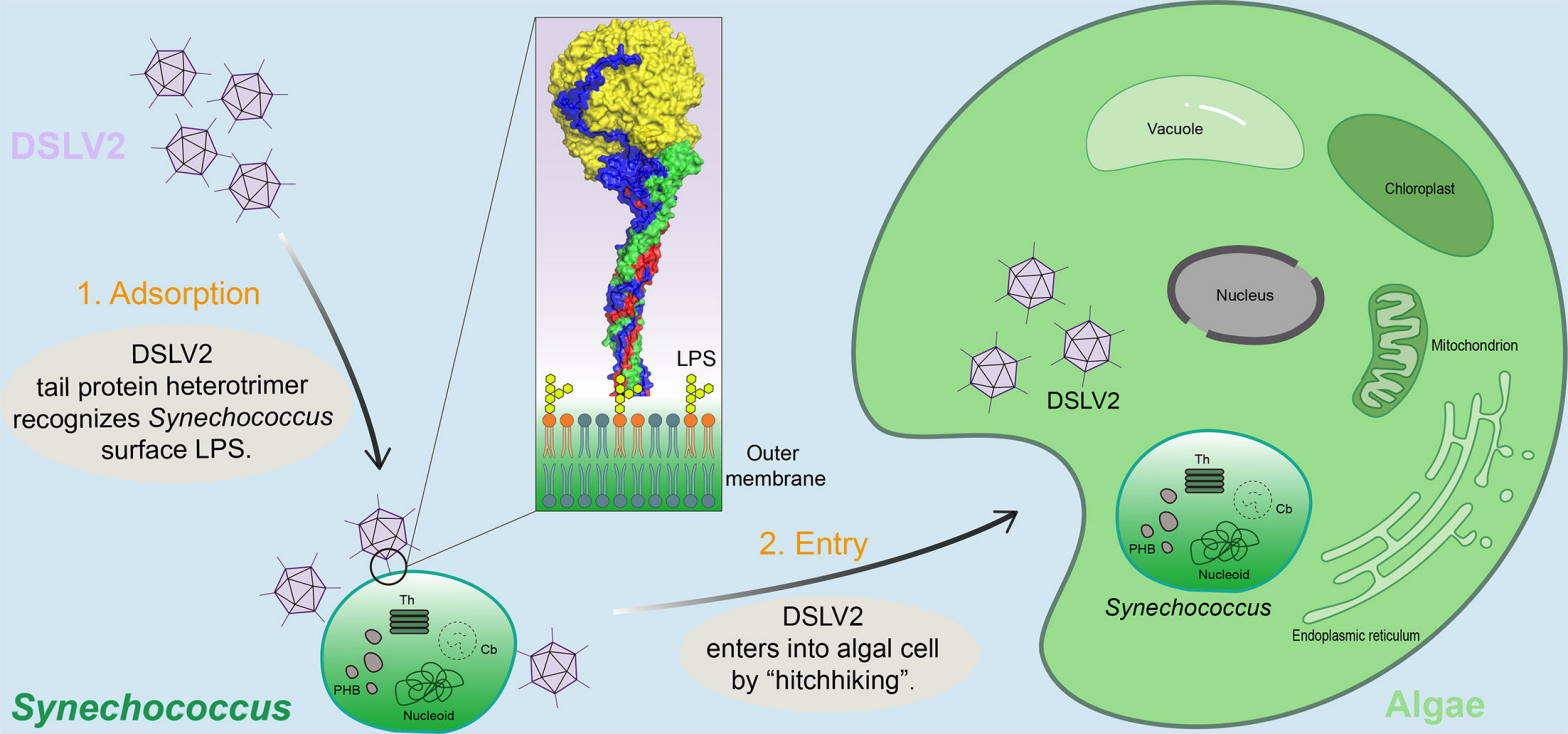
Proposed adsorption and cellular entry mechanism of DSLV2 virophage**.** (1) Recognition stage: the DSLV2 tail protein heterotrimer specifically binds to LPSs on *Synechococcus* cell surfaces, mediating viral attachment to these non-host cyanobacteria. (2) Hitchhiking entry: through bacterivorous feeding by unicellular eukaryotic algae, LPS-bound DSLV2 particles are internalized into algal cells via phagotrophic uptake. The three-dimensional structure in the magnified view (black circle) corresponds to the heterotrimeric tail protein of DSLV2 in Fig. 3C. Thylakoids (Th), carboxysomes (Cb), and polyhydroxybutyrate granules (PHB).

The DSLV2 virophage likely forms a CVv system with eukaryotic green algae as its host in Dishui Lake (19). In this ecosystem, *Synechococcus* dominates the bacterial community and occupies a shared niche with these algae (63), which have demonstrated predatory behavior toward *Synechococcus* (64–68). Accordingly, we propose that DSLV2 likely exhibits non-host adsorption to *Synechococcus* via LPS binding (Fig. 6E), while its eventual entry into algal cells may occur through viral hitchhiking mediated by algal bacterivory (Fig. 8). This hypothesis currently presents the most parsimonious explanation, combining ecological context with molecular evidence. Future studies could incorporate multiple experimental approaches to validate the DSLV2–*Synechococcus* interaction mechanism. First, high-resolution microscopic tracking of DSLV2 particles during algal bacterivory would provide visual evidence of viral entry dynamics. Second, comparative infection assays with and without bacterial intermediaries would help elucidate potential indirect infection routes. Notably, we would remain open to alternative or complementary life cycle mechanisms. For instance, DSLV2 might employ multiple entry strategies: (i) its tail proteins and/or MCP might recognize surface receptors on algae, analogous to the Mavirus-receptor interaction system (4, 8); or (ii) it could potentially interact with giant viruses (5, 7, 9) that co-infect the algal host, forming a tripartite infection complex. These possibilities warrant systematic investigation upon the successful establishment of the DSLV2-involved CVv co-culture model.

### Conclusion

Through systematic structural and functional characterization of DSLV2’s three consecutive tail proteins, we elucidate a novel mechanistic paradigm governing host entry in algal virophages. Our findings demonstrate that these tail proteins orchestrate a sophisticated two-step colonization strategy: (i) primary attachment to *Synechococcus* LPS receptors, followed by (ii) host entry mediated through algal bacterivory pathways. This insight resolves a fundamental gap in understanding the life cycle within the complex alga-giant virus-virophage tripartite infection system. Our findings fundamentally advance the comprehension of viral “hitchhiking” in aquatic ecosystems as well as provide a structural blueprint for further verifying the specific modulating interactions.

## Data Availability

The supplementary data are available on FigShare at https://figshare.com/s/7af50e7d73292a835549.
